# The Nordic Maintenance Care Program: Does psychological profile modify the treatment effect of a preventive manual therapy intervention? A secondary analysis of a pragmatic randomized controlled trial

**DOI:** 10.1371/journal.pone.0223349

**Published:** 2019-10-10

**Authors:** Andreas Eklund, Irene Jensen, Charlotte Leboeuf-Yde, Alice Kongsted, Mattias Jonsson, Peter Lövgren, Jakob Petersen-Klingberg, Christian Calvert, Iben Axén

**Affiliations:** 1 Karolinska Institutet, Institute of Environmental Medicine, Unit of Intervention and Implementation Research for Worker Health, Stockholm, Sweden; 2 Institute for Regional Health Research, University of Southern Denmark, Odense, Denmark; 3 Nordic Institute of Chiropractic and Clinical Biomechanics, Odense, Denmark; 4 Department of Sports Science and Clinical Biomechanics, University of Southern Denmark, Odense, Denmark; 5 Private practice, Lidköping, Sweden; 6 Private practice, Stockholm, Sweden; 7 Private practice, Borlänge, Sweden; 8 Private practice, Falkenberg, Sweden; Foundation IRCCS Neurological Institute C. Besta, ITALY

## Abstract

**Background:**

Chiropractic maintenance care is effective as secondary/tertiary prevention of non-specific low back pain (LBP), but the potential effect moderation by psychological characteristics is unknown. The objective was to investigate whether patients in specific psychological sub-groups had different responses to MC with regard to the total number of days with bothersome pain and the number of treatments.

**Method:**

Data from a two-arm randomized pragmatic multicenter trial with a 12-month follow up, designed to investigate the effectiveness of maintenance care, was used. Consecutive patients, 18–65 years of age, with recurrent and persistent LBP seeking chiropractic care with a good effect of the initial treatment were included. Eligible subjects were randomized to either maintenance care (prescheduled care) or to the control intervention, symptom-guided care. The primary outcome of the trial was the total number of days with bothersome LBP collected weekly for 12 months using an automated SMS system. Data used to classify patients according to psychological subgroups defined by the West Haven-Yale Multidimensional Pain Inventory (adaptive copers, interpersonally distressed and dysfunctional) were collected at the screening visit.

**Results:**

A total of 252 subjects were analyzed using a generalized estimating equations linear regression framework. Patients in the dysfunctional subgroup who received maintenance care reported fewer days with pain (-30.0; 95% CI: -36.6, -23.4) and equal number of treatments compared to the control intervention. In the adaptive coper subgroup, patients who received maintenance care reported more days with pain (10.7; 95% CI: 4.0, 17.5) and more treatments (3.9; 95% CI: 3.5, 4.2). Patients in the interpersonally distressed subgroup reported equal number of days with pain (-0.3; 95% CI: -8.7, 8.1) and more treatments (1.5; 95% CI: 0.9, 2.1) on maintenance care.

**Conclusions:**

Psychological and behavioral characteristics modify the effect of MC and should be considered when recommending long-term preventive management of patients with recurrent and persistent LBP.

## Introduction

Non-specific low back pain (LBP) is a highly prevalent condition, affecting a large part of the population with major consequences [1, 2]. For a highly disabling recurrent and costly condition it seems logical to invest in preventive strategies to mitigate and minimize its impact on the individual and on society [3–6]. However, the evidence for effective interventions aimed at preventing LBP is limited. To date, only exercise, exercise in combination with education and pre-planned manual treatment (chiropractic maintenance care, MC) have been shown to be effective [7, 8].

MC has traditionally been used by chiropractors and is described as a long-term management strategy, introduced when treatment benefit has been recorded after an initial care plan, with the aim of preventing future episodes and deterioration by treating the patient regularly irrespective of symptoms [9–14]. Ninety-eight percent of all Swedish chiropractors support the concept of MC and consider it to be a useful clinical procedure, at least in some circumstances [9]. MC is mainly used as a form of secondary or tertiary prevention aimed at recurrent and persistent conditions [9, 12, 15–17]. There seems to be a common patient-oriented management concept among chiropractors according to which patients are selected for MC mainly on the basis of their previous history of pain and the effectiveness of the initial care plan [10, 12, 15–20]. In previous studies, the proportion of chiropractic MC visits ranged between 14% and 41%. [10, 13, 14, 20–22] However, the evidence for its effectiveness and clinical usefulness have been lacking until recently [8, 11]. Previous research has been either efficacy studies or designed with little consideration of how MC is delivered in clinical practice [23–26].

In a comprehensive program starting 2008, the Nordic Maintenance Care Program, indications, content and frequency of MC have been systematically investigated by Scandinavian researchers [10, 12, 15–20]. Based on this knowledge, a randomized pragmatic clinical trial was designed to investigate the effectiveness of MC for recurrent and persistent LBP [27]. The trial found that the MC group had 12.8 fewer days with bothersome LBP (95% CI: 10.1, 15.5) over a year compared to the control group, who had treatments with a similar content but only when symptoms reappeared [8]. Although more effective, the number of visits was also somewhat higher in the MC group: 6.7 treatments (95% CI = 6.6, 6.8) compared to 4.8 treatments (95% CI = 4.7, 4.9) during the 52-week study period. A large variability in the outcomes within treatment groups suggested that there might be sub-groups of patients who responded better to MC than others.

Psychological [28, 29], behavioral [30] and social characteristics [31] of LBP patients are known to be important prognostic factors in the transition from acute to persistent pain states [32–35]. Based on the cognitive-behavioral conceptualization of pain, the Swedish version of the West Haven-Yale multidimensional pain inventory (MPI-S) has been shown to capture and measure the psychological and behavioral dimensions of the chronic pain experience [36].

As shown in pain populations for other interventions [37–39], outcomes can potentially be modified by psychological characteristics which can be identified by the MPI-S instrument. If so, this could lead to a tailored approach resulting in a greater effect of the MC intervention. Based on findings from previous research using different pain populations [40] we hypothesized that individuals classified as dysfunctional would benefit most from MC and patients classified as adaptive copers would benefit least.

The aim of the study was to explore the potential effect moderation of the psychological sub-groups (as identified by the MPI-S instrument) on MC.

The specific objectives were to investigate whether the MPI-S sub-groups had different outcomes in terms of total number of days with bothersome pain and total number of treatments during the 12-month study period.

## Materials and methods

### Trial design

This project is a secondary analysis of data from a pragmatic, investigator- and assessor- blinded randomized controlled trial with a two arm parallel design (Clinical trials.gov; NCT01539863 (February 22, 2012)). The trial has been described in detail in a published study protocol and the primary analysis has been reported in a recent publication [8, 27]. The trial was approved by the local ethics committee at Karolinska Institutet (2007/1458-31/4). Funding was awarded by the Institute for Chiropractic and Neuro-musculoskeletal Research, the European Chiropractors’ Union (project ID A13.02) and the Danish Chiropractic Research Foundation (grant number 11/148). This secondary analysis was funded by the European Center for Chiropractic Research Excellence (grant number 03-2016-SE/AE). None of the funding bodies had any influence on the design and execution of the project or on the analysis or interpretation of the data.

### Participants

Patients were recruited between 2012 and 2016 from 40 chiropractic clinics in Sweden, all part of a practice-based research network. Consecutive patients were screened when they visited a clinic for LBP, either a new complaint or a new episode of an old complaint. Eligibility-screening was executed in a three-stage process: at the first visit, at the fourth visit and at the inclusion visit.

At the first visit, patients were assessed for general eligibility according to the following criteria: aged 18–65; LBP with or without leg pain for more than 30 days during the previous year; a history of previous episodes of LBP; access to a mobile phone and the ability to send and receive SMS (text messages). They were excluded from the trial at this stage if they were pregnant, had received chiropractic treatment in the three previous months, were receiving completely subsidized treatment from a 3rd party (patients who didn’t pay anything to receive care), were suspected of having serious pathology (i.e. acute trauma, cancer, infection, cauda equina, osteoporosis, vertebral fractures) or other contraindications for manual therapy.

At the fourth visit, patients were assessed for improvement after the initial treatments. Only patients who reported a definite improvement (5^th^ level on a 5-step Likert scale) were included at this stage.

**Fig 1:**
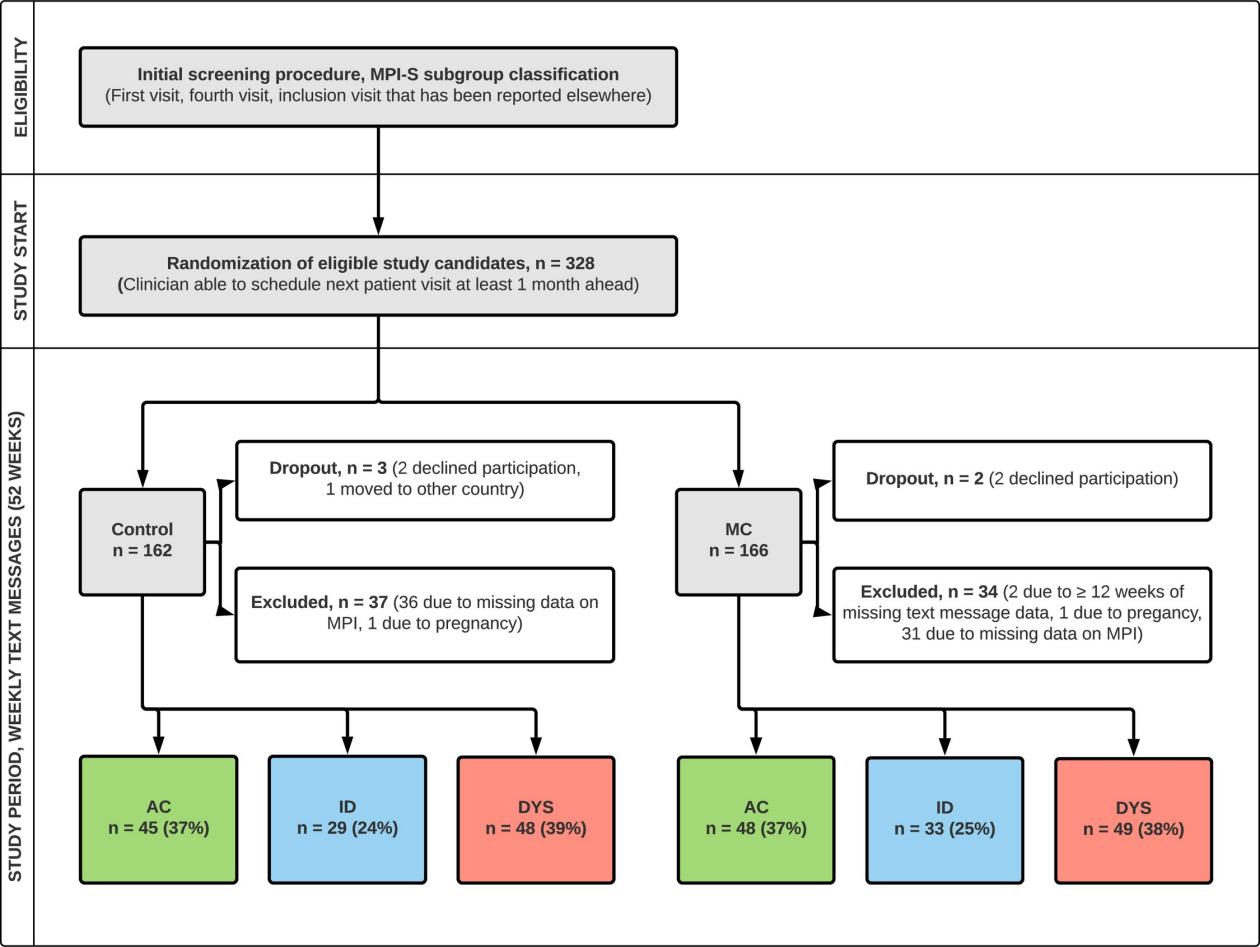
Patient flow. The initial screening procedure has been reported in a previous publication [8]; **MPI,** West Haven-Yale multidimensional pain inventory; **MC**, maintenance care; **AC**, adaptive copers; **ID**, interpersonally distressed; **DYS**, dysfunctional.

The study start was flexible in time depending on further improvement of the patient’s LBP after the fourth visit. The study started when the clinician considered the patient well enough to discontinue care or to be offered MC. At this study start visit, 328 patients were randomly allocated to MC or control. A flow chart in Fig 1 describes the inclusion procedure in detail.

### Interventions

Patients in MC programs are most commonly scheduled for visits at 1–3 month intervals, with continuous adjustments of the interval to achieve optimum effect with the longest possible period between visits. The treatment strategy is to manage musculoskeletal dysfunction and pain by means of manual therapy, individual exercise programs and lifestyle advice [41–46]. Based on previous research in the Nordic Maintenance Care Program, the two treatment arms in this trial were thought to represent two common strategies used in clinical practice [10–12, 15–20, 27]. Patients were randomized either to the MC strategy (i.e. pre-scheduled visits) with the aim of preventing future episodes, or to the control group, where patients were advised to contact their clinician when symptoms returned (symptom-guided treatment). In the MC group, the clinician scheduled visits at 1–3 month intervals depending on the clinical presentation. If the patient consulted for a new episode between the scheduled visits, they were treated accordingly (with more frequent visits) until ready to continue with the MC strategy. In the control group, when patients contacted their clinician upon return of symptoms, they were treated with frequent visits until again ready to discontinue care. For both groups, clinicians were instructed to tailor the treatment content and frequency of the acute treatment according to their usual practice. To achieve high compliance in both groups, the clinicians subsidized the treatment fee (50%) for all visits for treatment of the lumbar spine during the study period.

### Stratification by MPI-s sub-groups

At the first visit, patients completed the MPI-S instrument before the consultation with the chiropractor. The Swedish version of the instrument is a 34-item questionnaire converted into eight sub-scales divided into 2 parts. The first part consists of five scales designed to measure important dimensions of the chronic pain experience (pain severity (PS), interference (I), life control (LC), affective distress (AD), and support (S)). The second part consists of three scales assessing patients’ perceptions of how spouses or significant others respond to their pain behaviors and complaints (punishing responses (PR), solicitous responses (SR), and distracting responses (DR)) [36]. The original instrument was further developed to identify clusters/sub-groups [40] with similar characteristics that have been shown to be reliable, valid and useful in outcome-based research [47, 48]. Three distinctly different sub-groups have been identified: adaptive copers, interpersonally distressed and dysfunctional. These have been used in clinical settings to investigate neck pain and LBP [37–39], temporomandibular disorders [49], headaches [50], fibromyalgia [51] and cancer pain [52] and have been found to be associated with a number of different clinical outcomes.

Individuals in the adaptive copers group are characterized by low pain severity, low interference with everyday life, low life distress, a high activity level and a high perception of life control. This sub-group has the best prognosis and the lowest risk of long-term sick-leave [38, 53–55].

Individuals in the interpersonally distressed group are characterized by distrust of others whom they view as responsible for their problems. They consequently often feel that their spouses or significant others respond in a negative way to their pain behavior, for example not being supportive/helpful or expressing irritation, frustration and anger. The interpersonally distressed sub-group has been shown to have a poor prognosis and an increased risk of long-term sickness absence [38].

The dysfunctional sub-group is characterized by high pain severity, which interferes with everyday life, and by high affective distress, low perception of life control and low activity levels. This sub-group is most likely to rely on pain-avoidant coping strategies (e.g. catastrophizing) and to report more fear and avoidance of activities related to pain [40]. The dysfunctional sub-group has the worst prognosis and the highest risk of long-term sickness absence of the three sub-groups [38].

### Outcomes

The primary outcome of the trial was the total number of days with bothersome LBP over the 52-week period. In order to measure the impact of pain rather than the actual presence of pain, the term ‘bothersomeness’ has been used to capture the presence of consequential pain and has been recommended as a standard outcome measure in LBP outcome research [56–59]. Self-rated health [60], pain intensity [61], disability, prediction of work absence/healthcare consultations, and psychological distress (anxiety, depression) [62] have been found to correlate well with bothersomeness. To capture pain that was relevant to the patient over the entire study period, this outcome was used dichotomously, with the patient being asked whether they were bothered by their pain each day or not. Using the outcome this way is novel and the psychometric properties have only been tested in one previous study [61], which demonstrated a positive correlation between pain intensity and number of days with bothersome LBP.

Data were collected weekly using an automated SMS system (SMS-track) [63–65]. Patients were asked the following question: “*How many days during the previous week has your low back pain been bothersome (i*.*e*., *affected your daily activities or routines)*? *Please answer with a number between 0 and 7”*. If the patient failed to respond, a reminder was sent 48 hours later. If the patient didn´t respond to the reminder, a research assistant called the patient asking for a response. This strategy has been successful in previous projects and yielded a response rate of 98.9% in this RCT [8].

Baseline data and follow-up outcomes were collected from clinicians and patients throughout the inclusion procedure (first visit, fourth visit and study start) and at follow up (after completing the 52-week follow up period) [27]. In this secondary analysis, patient data include pain intensity (Numeric Rating Scale 0–10), self-rated health (EQ-5D), activity limitation (Roland Morris Disability Questionnaire), physical work demand (4-step categorical scale: physically heavy labor/interchanging between heavy and light/ standing and walking/sitting), self-reported sick-leave during the previous year (4-step scale: no/yes, 1–7 days in total/yes, 8–14 days in total/yes, ≥15 days in total), patient expectations of treatment outcome (Numeric Rating Scale 0–10: ranging from no change to very probable improvement), and clinician reported treatment content and number of visits (both during the initial inclusion and the entire study period) [27].

### Randomization and blinding

Using a randomization schedule, a statistician generated 40 permuted blocks containing 10 subjects in each with a 1:1 allocation ratio between groups. Consecutive sealed opaque envelopes with group assignment were opened (in a consecutive sequence) in front of the patient. The two treatment options were described to the patient as similar procedures, both being used in practice, not suggesting that either was more effective than the other. Clinicians were blinded up until the randomization procedure and investigators up until the completion of the primary data analysis, after which the allocation was revealed.

### Statistical methods

A similar statistical modelling strategy was used in this secondary analysis as in the primary analysis [8]. Data were reported with arithmetic means and 95% confidence intervals (95% CI) and the level of significance was set to 0.05. Subjects with ≥ 12 weeks of missing SMS data were excluded from the analysis.

The total number of days with bothersome LBP (sum) over 52 weeks was estimated using a generalized estimating equations (GEE) linear regression model, using an appropriate correlation structure and a robust variance estimator. QIC-values (quasi-likelihood under the independence model criterion), standard error and mean squared error were used to estimate the most appropriate correlation structure and model for the data. The statistical model was then tested for its predictive ability in a cross validation framework were the model was trained on the data from week 1–48 and tested on week 49–52. Initially, weekly estimates (mean and variance) were generated using the most appropriate GEE model, and in a second step these estimates were summarized for the entire 52 week study period to arrive at the primary outcome. The analysis was performed by considering treatment-group, time, number of days of bothersome LBP during week 1 of the trial (W1) and MPI-s sub-groups as covariates in the model. These covariates were considered for the analysis a-priori based on the procedure used in the original effect evaluation [8]. A best subset regression procedure was used to arrive at a final statistical model for the included covariates in the analysis. All covariates were included in the model individually as well as interaction terms, and were excluded based on p-value and goodness of fit estimates. A significant (p < 0.001) 4-way interaction term was present (‘Treatment-group’, ‘MPI sub-group’, ‘W1 (week 1)’ and ‘Time’), described in a supplementary file (S1 Table). The analysis was therefore performed in a second step with the MPI-S sub-groups as separate strata. This reduced the complexity of the analytical strategy and allowed for individual modelling strategies for each MPI-S sub-group. The best fit for the pain data (same for all MPI-S sub-groups) could be estimated with a normal distributed M-dependent (1) model with the following covariates: ‘Treatment-group’, ‘Time’, ‘Time*Time’, ‘W1’ and ‘Treatment-group*W1*Time’.

The total number of visits was estimated with a GEE Poisson regression model, using the appropriate correlation structure and a robust variance estimator. The analysis of visit data followed a similar analytical strategy as the analysis of number of days with bothersome LBP, by considering treatment-group, time, W1 and MPI sub-groups as covariates in the model. The best fit for the visit data could be estimated individually for each MPI sub-group stratum; adaptive coper: Poisson distributed M-dependent (5) model with the covariate structure: ‘Treatment-group’, ‘Time’, ‘W1’ and ‘W1*Time*Time’; interpersonally distressed: Poisson distributed M-dependent (7) model with the covariate structure: ‘Treatment-group’, ‘Time’, and ‘W1’; dysfunctional: Poisson distributed M-dependent (8) model with the covariates: ‘Treatment-group’, ‘Time’, ‘W1’ and ‘Treatment-group*Time’. Five missing data points for the variable W1 (prediction variable) was imputed using the next value (week 2) carried backward to avoid losing subjects in the analysis. No imputation was made for the rest of the dataset (outcome variables) given the flexible nature of the GEE model as all data are used in the estimating process.

All analyses were performed using the statistical software SPSS version 25 [66].

## Results

### Patient flow

Of the 328 subjects who were randomized into the trial, five dropped out after inclusion and two were excluded due to pregnancy. A total of 69 subjects were excluded from the analysis because of missing data, leaving 252 subjects (77% of the eligible subjects) in the final analysis. Fig 1 describes the patient flow in detail.

### Descriptive data

With the exception of one variable, there were no statistically significant differences for the descriptive data (reported in Table 1 and in S2 Table) between treatment arms (MC and control) in each MPI sub-group and no obvious systematic bias was observed. In the interpersonally distressed sub-group (n = 62), the MC treatment-group reported 1.2 (p = 0.019) more days with bothersome LBP than the control group during the first week of the study period. No such difference was observed in the adaptive coper (n = 93) or dysfunctional (n = 97) sub-groups. The division into the MPI-S sub-groups clearly differentiated health characteristics: individuals in the interpersonally distressed and dysfunctional sub-groups demonstrated higher pain intensity, activity limitation (RMDQ) and lower self-rated health (EQ-5D).

**Table 1:** Descriptive information.

Variable ^A^		MPI sub-group					
		Adaptive coper		Interpersonally distressed		Dysfunctional	
		**MC** **n = 48**	**Control** **n = 45**	**MC** **n = 33**	**Control** **n = 29**	**MC** **n = 49**	**Control** **n = 48**
Age at study start, mean (SD)		44.8 (10.4)	43.6 (12.6)	43.8 (10.1)	41.3 (12.8)	41.7 (12.6)	45.8 (11.5)
Female, % (n)		61.7 (29)	62.8 (27)	65.6 (21)	63.0 (17)	59.6 (28)	56.5 (26)
Type of work, % (n)	Physically heavy	6.7 (3)	6.7 (3)	12.1 (4)	24.1 (7)	16.3 (8)	16.7 (8)
	Intermittent heavy/light	37.8 (17)	37.8 (17)	30.3 (10)	24.1 (7)	44.9 (22)	37.5 (18)
	Walking/standing	20.0 (9)	20.0 (9)	42.4 (14)	27.6 (8)	32.6 (16)	43.7 (21)
	Sitting	62.5 (25)	62.5 (25)	54.5 (18)	44.8 (13)	38.8 (19)	35.4 (17)
Pain in the thigh and lower leg, % (n)		25.0 (12)	13.3 (6)	33.3 (11)	27.6 (8)	18.4 (9)	20.8 (10)
Pain in the neck and/or thoracic spine, % (n)		60.4 (29)	71.1 (32)	75.8 (25)	79.3 (23)	85.7 (42)	70.8 (34)
Pain intensity, 0–10, mean (SD)	1st visit	4.3 (1.8)	4.3 (1.8)	6.1 (2.0)	5.7 (1.7)	5.8 (1.7)	6.2 (1.7)
	4th visit	1.7 (1.3)	1.7 (1.3)	2.8 (1.6)	2.2 (1.2)	2.0 (1.1)	2.3 (1.8)
	Study start	1.3 (1.1)	1.3 (1.1)	2.2 (1.4)	2.3 (1.4)	2.0 (1.1)	2.3 (1.8)
EQ-5D score (study start) 0–1, mean (SD)		0.81 (0.03)	0.79 (0.11)	0.59 (0.24)	0.67 (0.22)	0.59 (0.24)	0.67 (0.19)
RMDQ Score (study start), mean (SD)		3.0 (3.0)	3.1 (4.3)	6.0 (3.9)	4.1 (3.0)	6.1 (4.3)	5.2 (3.5)
Number of treatments during initial period, mean (SD)		5.4 (2.6)	6.2 (2.8)	5.7 (1.8)	6.5 (2.5)	6.4 (2.6)	6.4 (3.0)

^**A**^, No imputation has been made for missing data, mean values and percentages are based on true responses for each variable; MPI, West Haven-Yale multidimensional pain inventory; **MC**, maintenance care; **SD**, standard deviation; **n**, number of subjects; **RMDQ**, Roland Morris disability questionnaire.

### Outcomes

**Fig 2:**
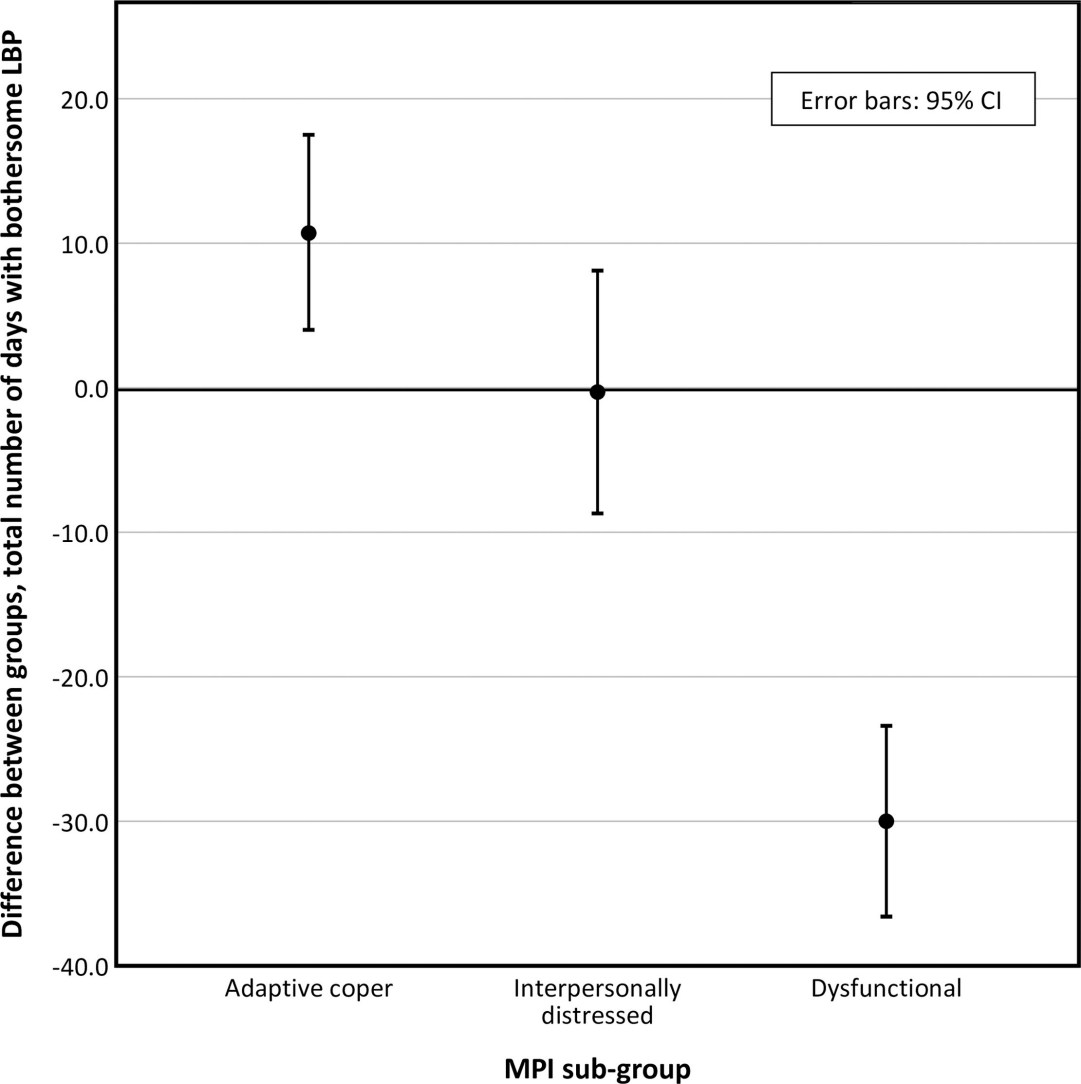
Total number of days with bothersome low back pain, differences between groups (MC-Control). **MC**; maintenance care; **CI**, confidence interval; **LBP**, low back pain; **MPI**, West Haven-Yale multidimensional pain inventory.

A large positive effect of MC was observed in the dysfunctional sub-group. No effect was observed in the interpersonally distressed sub-group and a negative effect was observed in the adaptive coper sub-group (Table 2 and Fig 2).

**Table 2:** Total number of days with bothersome low back pain, group estimates and differences between groups (MC-Control).

MPI sub-group	Group	Total number of days with bothersome LBP, estimate (95% CI)	SE	p
**Adaptive coper**	MC	78.3 (73.3, 83.4)	2.595	-
	Control	67.6 (63.2, 72.1)	2.274	-
	Difference	10.7 (4.0, 17.5)	3.450	0.002
**Interpersonally distressed**	MC	94.4 (89.9, 98.9)	2.301	-
	Control	94.7 (87.7, 101.8)	3.604	-
	Difference	-0.3 (-8.7, 8.1)	4.706	0.944
**Dysfunctional**	MC	79.5 (75.2, 83.8)	2.178	-
	Control	109.5 (104.4, 114.5)	2.567	-
	Difference	-30.0 (-36.6, -23.4)	3.367	<0.001

**CI**, confidence interval; **SE**, standard error of the mean; **p**, p-value; **MC**, maintenance care; **MPI**, West Haven-Yale multidimensional pain inventory; **LBP**, low back pain.

**Fig 3:**
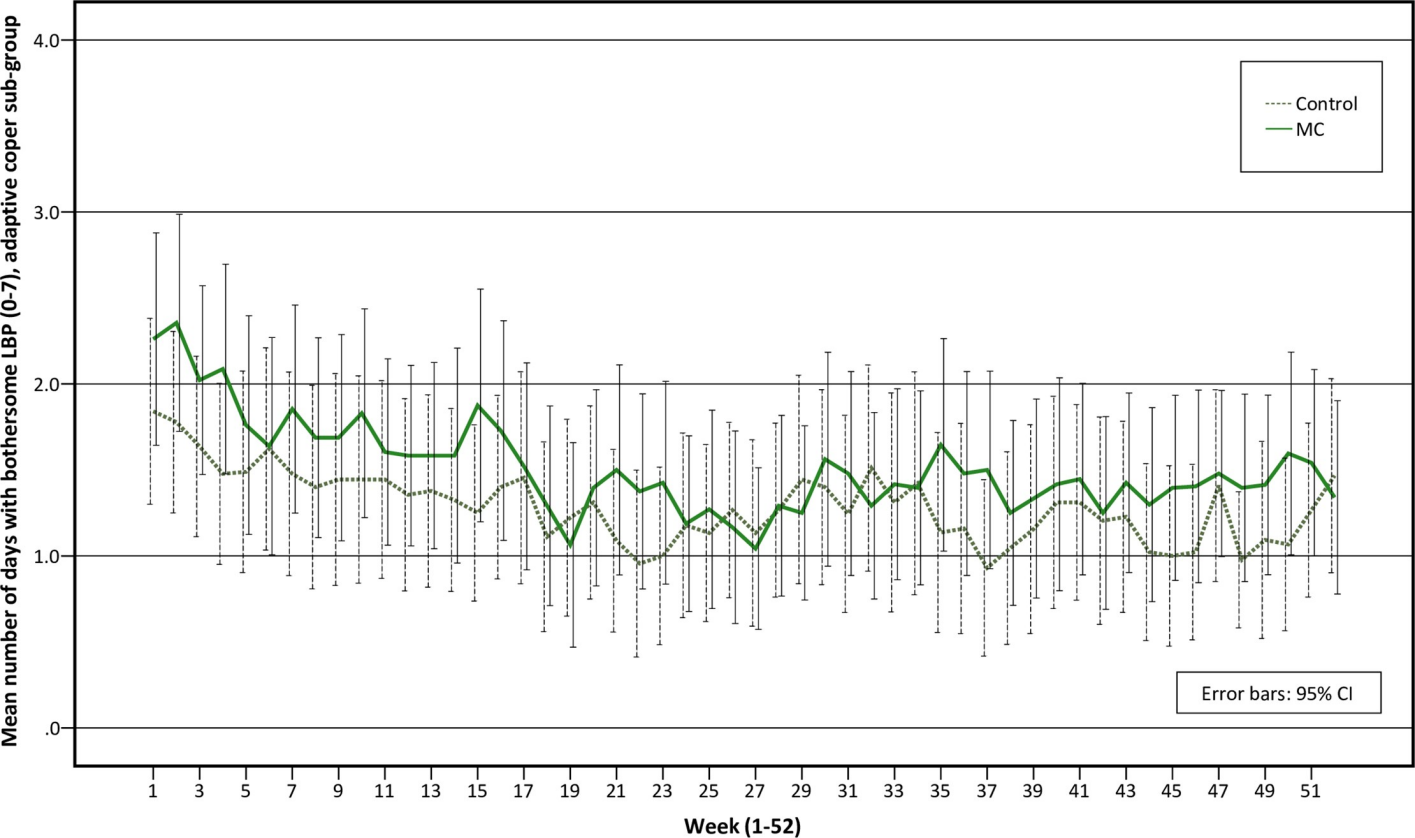
Pain trajectories for treatment groups in the adaptive coper sub-group.

**Fig 4:**
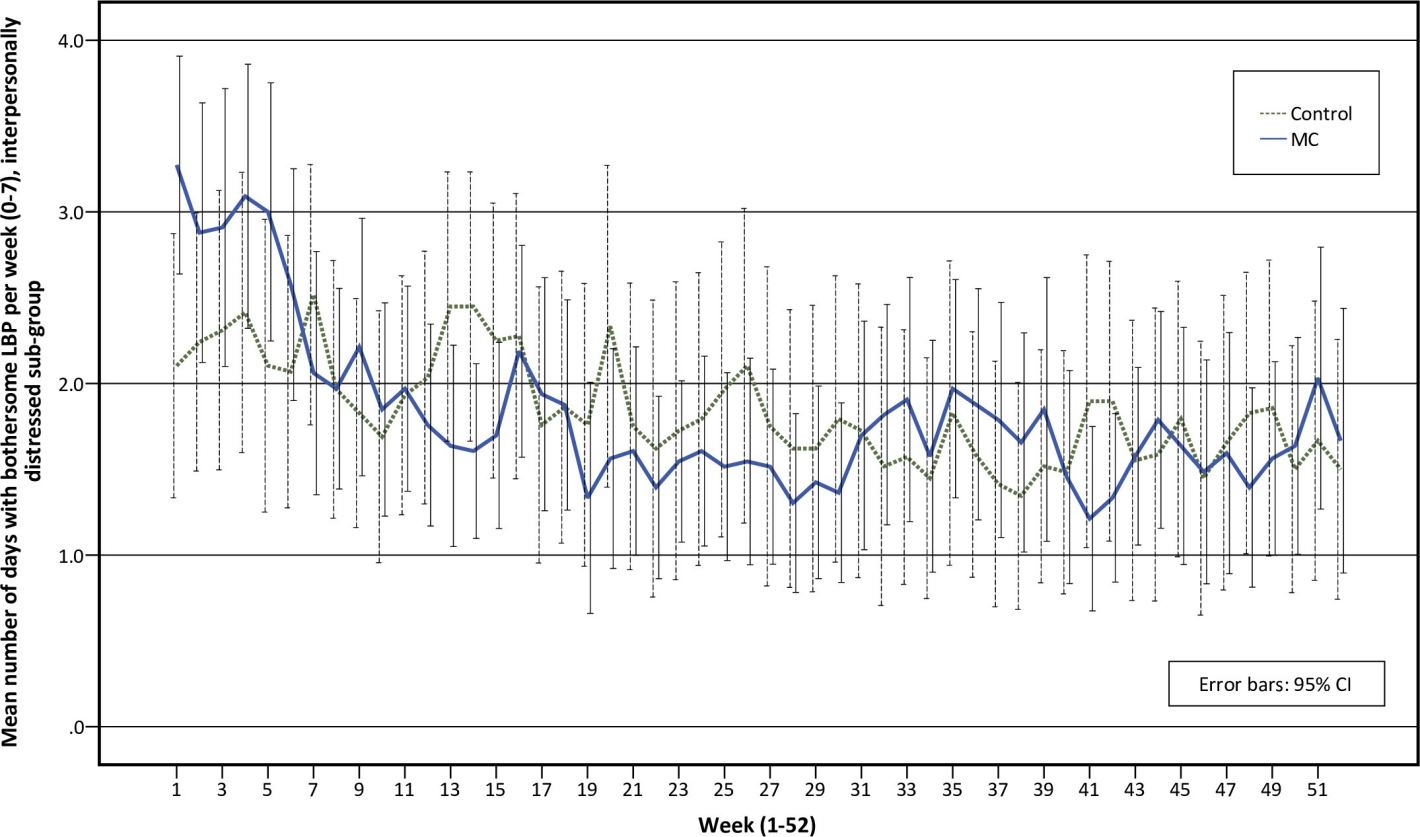
Pain trajectories for treatment groups in the interpersonally distressed sub-group.

**Fig 5:**
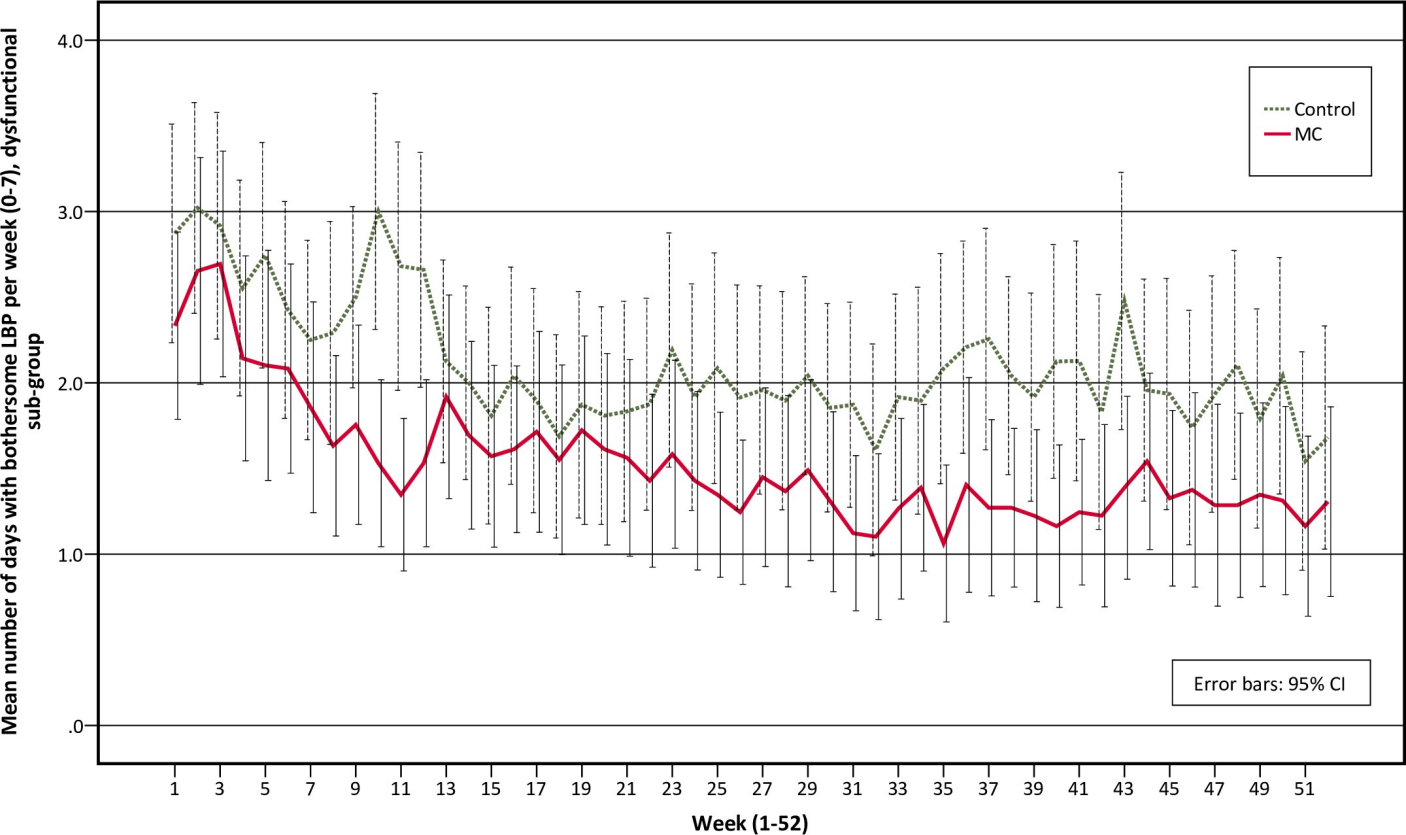
Pain trajectories for treatment groups in the dysfunctional sub-group.

**Fig 6:**
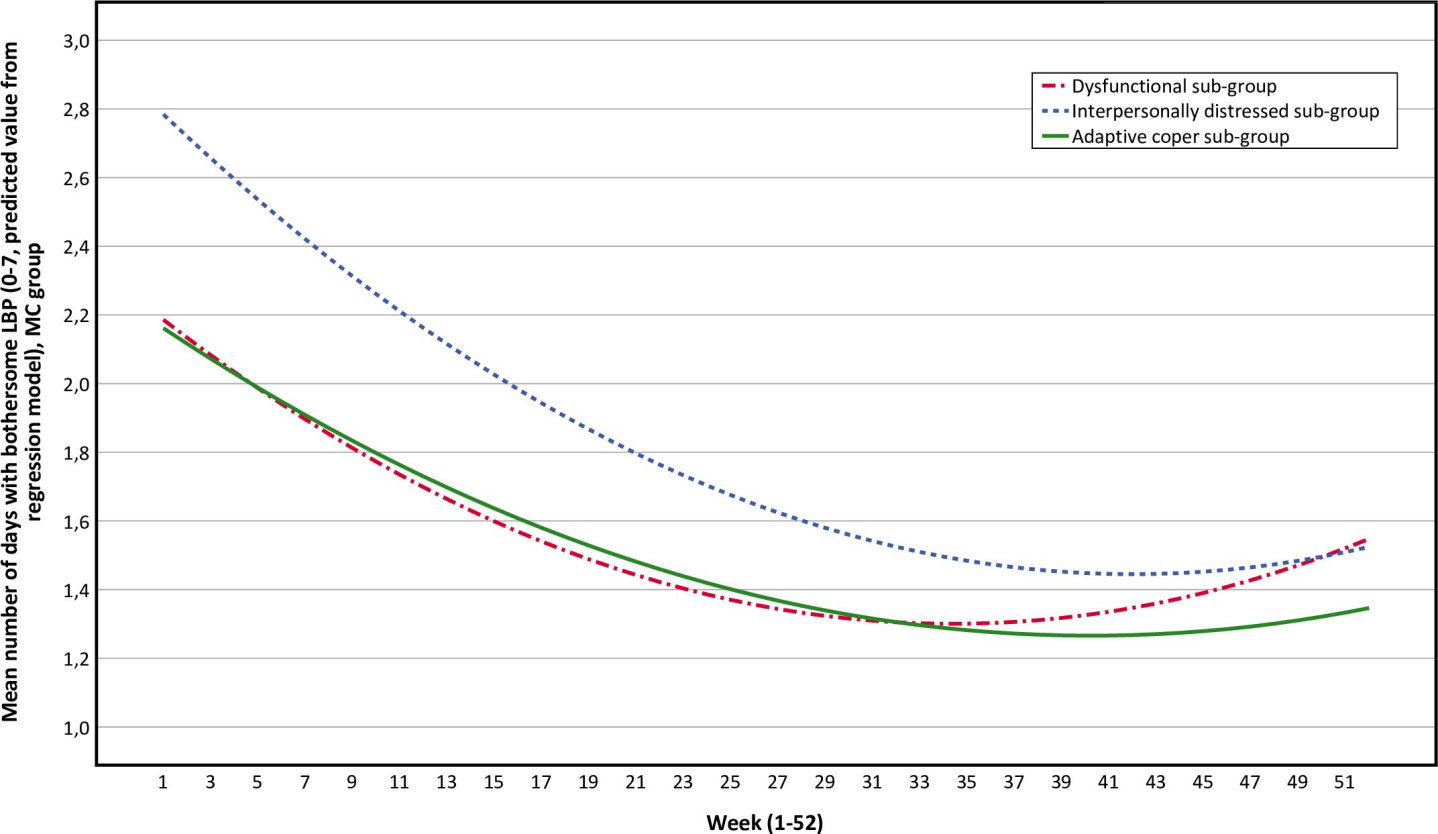
Pain trajectories for the maintenance care group (all sub-groups).

**Fig 7:**
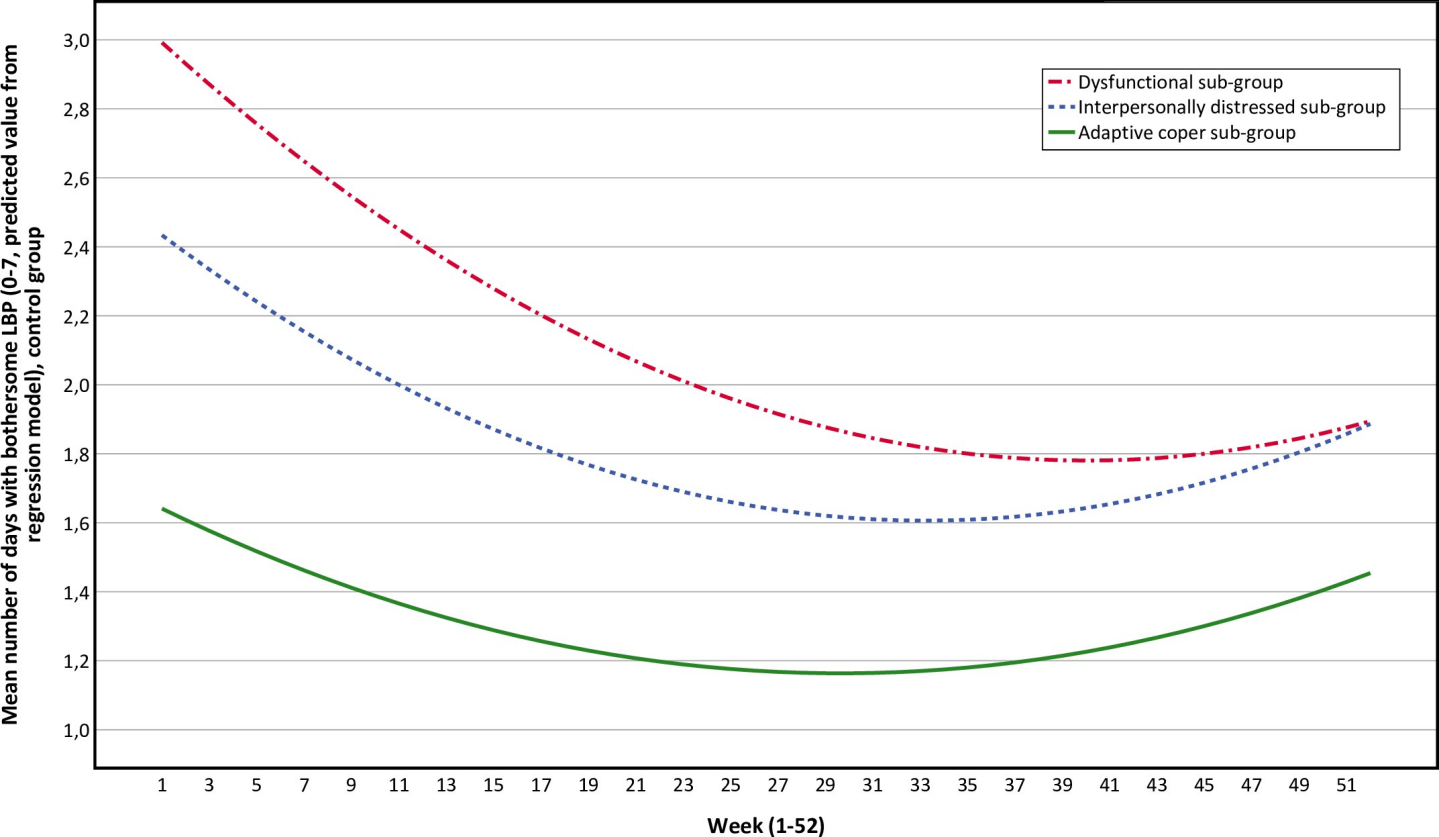
Pain trajectories for the control group (all sub-groups).

The adaptive coper and interpersonally distressed sub-groups follow similar pain trajectories over time, with small to no differences between the MC group and the control group over time (Figs 3 and 4). The pain trajectories for the dysfunctional sub-group clearly separates treatment groups with a stable trend over time, with the control group reporting more days with bothersome LBP than the MC group (Fig 5). To illustrate the effect modification from the psychological profile further, data has been plotted as graphs with all psychological subgroups together for the control and MC groups respectively (Fig 6 and Fig 7). The data show that the dysfunctional sub-group show a very similar pain trajectory to the adaptive coper sub-group for patients receiving MC whereas for the control group, the differences are large between the psychological sub-groups.

**Fig 8:**
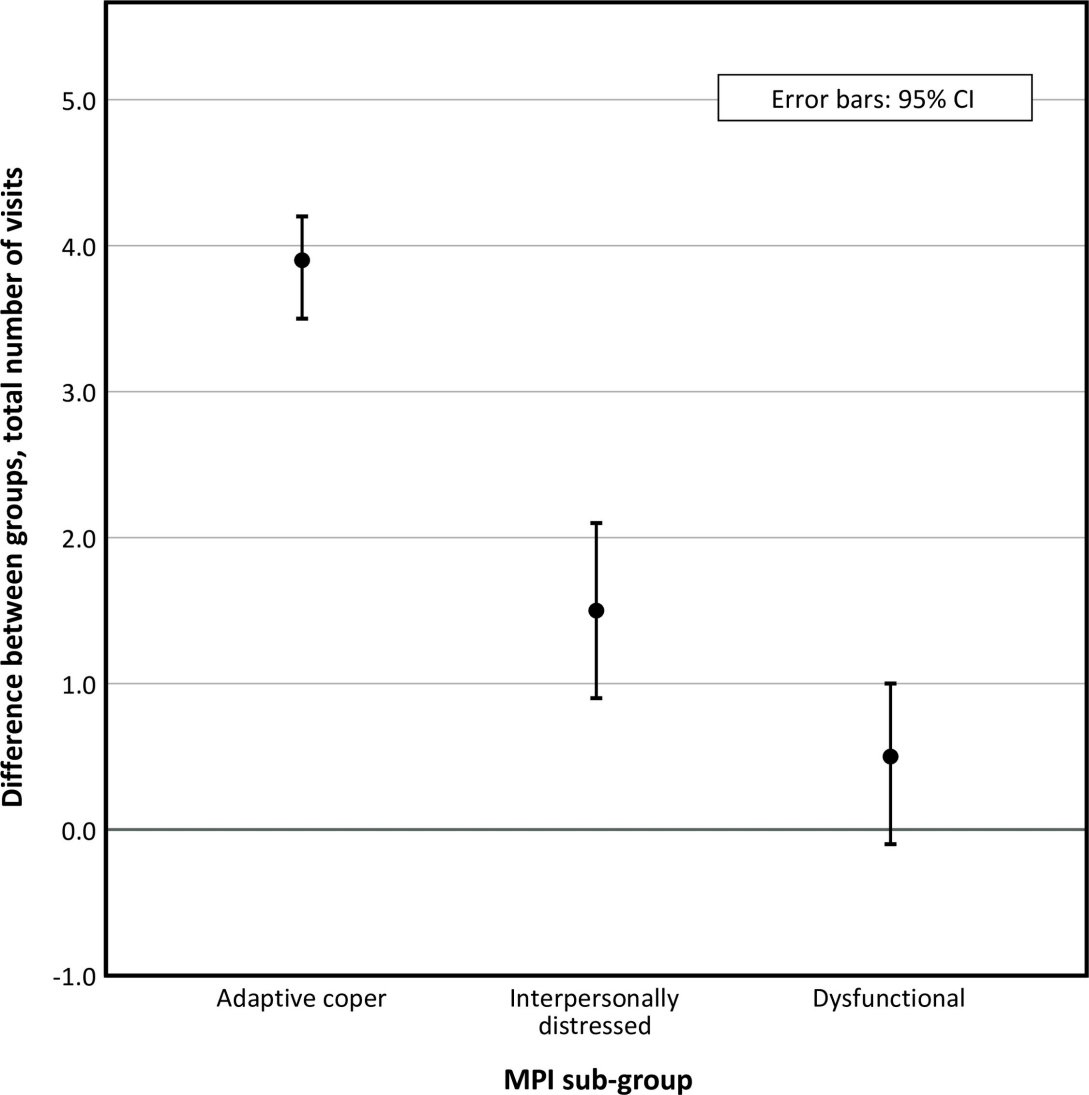
Total number of visits during study period, group differences (MC-control). **MC**; maintenance care; **CI**, confidence interval; **LBP**, low back pain; **MPI**, West Haven-Yale multidimensional pain inventory.

A different pattern is revealed for number of visits. Patients in the adaptive coper and interpersonally distressed sub-groups who received MC reported more visits (3.9 and 1.5 respectively) than patients in the control groups, whereas in the dysfunctional sub-group there was no difference in number of visits between the MC and control group. The visit data are presented in Table 3 and Fig 8.

**Table 3:** Total number of visits during study period, group estimates and group differences (MC-control).

MPI sub-group	Group	Total number of visits, estimate (95% CI)	SE	P
**Adaptive coper**	MC	7.0 (6.8, 7.3)	0.140	-
	Control	3.2 (2.9, 3.4)	0.135	-
	Difference	3.9 (3.5, 4.2)	0.194	<0.001
**Interpersonally distressed**	MC	6.9 (6.5, 7.4)	0.214	-
	Control	5.4 (5.0, 5.9)	0.230	-
	Difference	1.5 (0.9, 2.1)	0.314	<0.001
**Dysfunctional**	MC	7.3 (7.0, 7.7)	0.168	-
	Control	6.9 (6.4, 7.3)	0.238	-
	Difference	0.5 (-0.1, 1.0)	0.292	0.118

**CI**, confidence interval; **SE**, standard error of the mean; **p**, p-value; **MC**, maintenance care.

In the S3 Table additional follow-up data are reported for all three MPI-S sub-groups. Most outcomes were quite similar between MC and control within each MPI-S sub-group. Some potential between-group differences were noted (treatment content, treatment by other health professional, patient satisfaction), however these were not statistically significant with few individuals in each groups, and should be interpreted with much caution.

## Discussion

This is the first study to investigate the effectiveness of a preventive manual treatment protocol on the number of days with bothersome pain in patients within different psychological sub-groups.

As we hypothesized, patients most bothered by symptoms and with a less favorable psychological profile (the dysfunctional sub-group) reported better outcomes from the MC approach. The effect is large and clinically relevant, with 30 less days with bothersome (activity limiting) LBP over 12 months. Surprisingly, this effect was achieved with the same number of visits as with symptom-guided treatment (control). MC had a negative effect among the least affected patients (the adaptive coper sub-group) who also received a higher number of visits than the control group. The difference in effect was similar between MC and control in the interpersonally distressed sub-group, but the number of visits was slightly higher for the MC treatment group.

The data from this trial makes a compelling argument for informing the way MC is delivered in clinical practice. We can now identify a sub-group of patients, the dysfunctional sub-group, for whom MC appears to be more effective than symptom-guided treatment with the same number of visits. The results also suggest that MC should not be recommended for another specific sub-group of patients, the adaptive coper sub-group: it is counterproductive and a symptom-guided approach would be more suitable. The findings for the interpersonally distressed sub-group are more ambiguous. However, it is reasonable to suggest that either MC or symptom-guided treatment could be offered as the difference in the number of visits is small with similar effects between the groups.

The data from this trial is a welcome contribution to the literature for all clinicians who see patients with LBP in primary care. It will also be important for the debate within the manual professions concerning the long-term management of musculoskeletal disorders. If these findings were extrapolated into a broader clinical context, MC would primarily be recommended for individuals with the “worst” clinical picture, i.e. reporting high levels of pain, marked interference with everyday life, high affective distress, low perception of life control and low activity levels. These individuals could possibly also benefit from an exercise-based intervention within a bio-psycho-social framework, although they are very likely to show low compliance with such strategies [67]. Previous research has shown that LBP patients classified as dysfunctional have a better effect on activity limitation and pain from comprehensive treatment packages including a combination of manipulation, exercise and physician consultation compared to the adaptive coper and interpersonally distressed subgroups [67]. It has been hypothesized that pain-related anxiety and decreased acceptance of pain may contribute to the dysfunctional patients sensitivity to treatment [67]. Several studies have investigated different pain conditions and sick leave across MPI-S subgroups [37, 38, 68, 69]. There seem to be a differentiated treatment response where the adaptive coper sub-group are more likely to respond well to single unidimensional treatments [37, 38, 68–70].

Due to the pragmatic design of the trial it is difficult to draw conclusions about the potential mechanisms of effect in the dysfunctional sub-group or the lack of effect in the adaptive coper and interpersonally distressed sub-groups. It is possible that the structure of the preplanned visits creates a safe experience in which reassurance and support from the chiropractor allows the patients in the dysfunctional sub-group to explore and challenge their pain-avoidant coping strategies (e.g. catastrophizing), resulting in less fear and avoidance. By knowing that they soon will see their clinician the patient may be able to “risk a relapse” and therefore dare to expose themselves to pain and painful situations. In fact, longitudinal data from the United Kingdom have shown that fear avoidance beliefs, catastrophizing and self-efficacy may improve significantly within a few days of a visit to a chiropractor [46]. The adaptive coper sub-group, on the other hand, may be unnecessarily reminded of their problem by the preplanned visits, which might increase fear and avoidance. This possible explanation is intriguing but we lack data to support such conclusions.

There was no statistically significant difference in treatment content between the MPI-S subgroups. As described above, one may speculate that a more comprehensive intervention would benefit the dysfunctional sub-group. However, such results were found after multimodal interventions including combined physical and psychological components provided by multiple therapists. In this trial, patients were treated by a single clinician/chiropractor and the data describing treatment content are not suggesting such differences, but this question remains and needs to be tested in future studies.

The fact that clinicians were not blinded to the treatment assignment, even though instructed to behave the same towards all patients, may still have resulted in different behaviors and procedures within each of the two treatment arms. Previous research have shown that that clinician behavior may change and result in a systematic bias with regards to the interaction with the patient in an un-blinded procedure [71, 72]. Although the treatment assignments was known to the clinicians, they were completely naïve to the psychological status and subgroup assignment as the method for the subgroup analysis was not presented or discussed prior to the study. It is unclear to what extent clinician behavior may have affected the outcome, however, it cannot be ruled out as potential confounder when interpreting the results.

A weakness of the study is that the trial was not primarily designed for the sub-group analysis. This results in a theoretically underpowered design, subject to bias from random error. As a result, secondary analyses are generally considered to be hypothesis-generating rather than confirming given the limitations with regards to statistical power and design in general. However, the random allocation seems to have yielded similar groups in terms of descriptive baseline data and no systematic bias can be observed. The estimates of the primary outcome are robust with relatively small variations in each sub-group. This is demonstrated by the narrow 95% confidence intervals as a consequence of the high-frequency repeated individual data, and should be considered reliable and trustworthy. Given the underpowered design, the research group was surprised to find such large effect sizes in the adaptive coper and dysfunctional sub-groups with such narrow confidence limits. It is unlikely these findings would change much with a larger sample and although this is a secondary sub-group analysis.

All patients in the trial received a 50% subsidized fee for treatments during the study period. Potentially, the lower fee may have resulted in patients seeking more treatment than they normally would have done. This may have overestimated the number of visits, in particular in the symptom guided control group where participants were in control of the treatment frequency.

The major strengths of the study are the original data set of high quality, the collection of sub-group data prior to randomization, the delivery of treatment without access to sub-group information and the long follow up period with high-frequency repeated data that allows for a detailed analysis of the pain trajectories for each sub-group. Further, this trial makes use of all the available evidence in the field concerning how MC is used. We therefore consider the results to be robust and clinically relevant. In order to effectively implement the patient selection procedure described in this paper, a quick and clinically applicable way of subgrouping patients (like a short form of the MPI-S instrument) needs to be developed to be used in a busy clinical practice. When such an instrument has been developed to guide the selection of patients, the procedure may easily be transferred to clinical practice as the actual MC concept is already in place in many chiropractic clinics as reported in the publications from the Nordic Maintenance Care program [9, 10, 12, 15–20].

Although the results from this trial are robust and compelling, the data need verification in other populations. Further research is needed to investigate the effectiveness of MC in pediatric, elderly and pregnant populations as well as exploring the underlying mechanisms more carefully. Questions like “Does MC work by reducing the number of episodes, reducing the number of days or intensity of each episode or by an overall reduction of pain during the entire study period?” needs to be answered. If MC is effective as a means of secondary prevention it would mean that the time to or between new episodes would be longer or/and that the flare-up would be less severe. The longitudinal dataset from this trial allows for detailed analysis of pain trajectories of the periods around the visits, which may reveal how MC effect pain free periods and flare-ups.

Another logical next step would be to reproduce the overall method in another pragmatic clinical trial primarily aimed at investigating cost-effectiveness in a dysfunctional population without any financial reimbursement. Future research should apply these findings as part of an implementation strategy with the aim of improving clinical outcomes and promoting an evidence-based approach to MC.

## Conclusion

Psychological characteristics appears to modify the effect of MC and should be taken into consideration in the long-term management of patients with recurrent and persistent LBP. Patients who show a favorable response to an initial course of chiropractic care should be considered for MC if they report high pain severity, marked interference with everyday life due to pain, high affective distress, low perception of life control and low activity levels at baseline. Patients who, on the other hand, report low pain severity, low interference with everyday life due to pain, low life distress, high activity levels and a high perception of life control should probably not be recommended MC and instead only receive care when they experience a relapse of pain.

## Supporting information

S1 Table Description of interaction term. **Time**, week = 1, 2, 3, …, 52.; **W1**, number of days with bothersome low back pain week 1 of the study period = 0, 1, …, 7.; **AC**, adaptive copers; **ID**, interpersonally distressed; **DYS**, dysfunctional.
(DOCX)

S2 Table Additional descriptive data. ^**A**^, No imputation has been made for missing data, mean values and percentages are based on true responses for each variable; **MPI,** West Haven–Yale Multidimensional Pain Inventory; **MC**, maintenance care; **MOB**, mobilization; **ACT**, mechanically assisted spinal manipulative therapy using the activator instrument or similar; **DROP**, mechanically assisted spinal manipulative therapy using a drop mechanism in table; **STT**, soft tissue treatment; **ATM**, use of active therapeutic movement treatment table; **SD**, standard deviation; **n**, number of subjects; *,p-value for the difference = 0.019.
(DOCX)

S3 Table Additional follow-up data. ^**A**^, no imputation has been made for missing data, mean values and percentages are based on true responses for each variable; **MPI,** West Haven–Yale Multidimensional Pain Inventory; **MC**, maintenance care; **MOB**, mobilization; **ACT**, mechanically assisted spinal manipulative therapy using the activator instrument or similar; **DROP**, mechanically assisted spinal manipulative therapy using a drop mechanism in table; **STT**, soft tissue treatment; **ATM**, use of active therapeutic movement treatment table; **SD**, standard deviation; **n**, number of subjects; **RMDQ**, Roland Morris disability questionnaire.
(DOCX)

## Abbreviations

LBP: low back pain
MC: maintenance care
RCT: randomized controlled trial
SMS: text message
95% CI: 95% confidence intervals
LKR: Swedish chiropractic association
MPI: West-Haven Yale multidimensional pain inventory
AC: adaptive coper
ID: Interpersonally distressed
DYS: dysfunctional
RMDQ: Roland Morris disability questionnaire
SMT: spinal manipulative therapy
MOB: mobilization
ACT: mechanically assisted spinal manipulative therapy using the activator instrument
DROP: mechanically assisted spinal manipulative therapy using a drop mechanism in table
STT: soft tissue therapy
ATM: use of active therapeutic movement treatment table
SD: standard deviation
EQ5D: EuroQol 5 dimensions
p: p-value
GEE: generalized estimating equations
QIC: quasi-likelihood criterion
