# Supplementary material for: The Nordic Maintenance Care Program: Does psychological profile modify the treatment effect of a preventive manual therapy intervention? A secondary analysis of a pragmatic randomized controlled trial
Source: PLoS One. 2019 Oct 10;14(10):e0223349. doi: 10.1371/journal.pone.0223349 (PMC6786625; doi:10.1371/journal.pone.0223349)
Supplement: S1 Table — Time, week = 1, 2, 3, …, 52.; W1, number of days with bothersome low back pain week 1 of the study period = 0, 1, …, 7.; AC, adaptive copers; ID, interpersonally distressed; DYS, dysfunctional. (DOCX) [file pone.0223349.s001.docx]

| **Interaction term** | **W1 mean** | **Estimate** | **p-value** |
| --- | --- | --- | --- |
| [**Treatment-group** = Contol (0)] * [**MPI sub-group** = ID] * [**Time**] * [**W1**] | 1.84 | -0.00676 | 0.116 |
| [**Treatment-group** = Contol (0)] * [**MPI sub-group** = DYS] * [**Time**] * [**W1**] | 2.11 | -0.00794 | <0.001 |
| [**Treatment-group**  = Contol (0)] * [**MPI sub-group** = AC] * [**Time**] * [**W1**] | 1.29 | -0.00816 | 0.003 |
| [**Treatment-group** = Intervention (1)] * [**MPI sub-group** = ID] * [**Time**] * [**W1**] | 1.82 | -0.01010 | <0.001 |
| [Treatment-group = Intervention (1)] * [**MPI sub-group** = DYS] * [**Time**] * [**W1**] | 1.53 | -0.00821 | 0.001 |
| [**Treatment-group** = Intervention (1)] * [**MPI sub-group** = AC] * [**Time**] * [**W1**] | 1.51 | -0.00725 | 0.010 |
