# Supplementary material for: The Nordic Maintenance Care Program: Does psychological profile modify the treatment effect of a preventive manual therapy intervention? A secondary analysis of a pragmatic randomized controlled trial
Source: PLoS One. 2019 Oct 10;14(10):e0223349. doi: 10.1371/journal.pone.0223349 (PMC6786625; doi:10.1371/journal.pone.0223349)
Supplement: S2 Table — A, No imputation has been made for missing data, mean values and percentages are based on true responses for each variable; MPI, West Haven–Yale Multidimensional Pain Inventory; MC, maintenance care; MOB, mobilization; ACT, mechanically assisted spinal manipulative therapy using the activator instrument or similar; DROP, mechanically assisted spinal manipulative therapy using a drop mechanism in table; STT, soft tissue treatment; ATM, use of active therapeutic movement treatment table; SD, standard deviation; n, number of subjects; *,p-value for the difference = 0.019. (DOCX) [file pone.0223349.s002.docx]

| **Variable^A^** | | **MPI sub-group** | | | | | |
| --- | --- | --- | --- | --- | --- | --- | --- |
|  |  | **Adaptive coper** | | **Interpersonally distressed** | | **Dysfunctional** | |
|  | | **MC**  **n=48** | **Control**  **n=45** | **MC**  **n=33** | **Control**  **n=29** | **MC**  **n=49** | **Control**  **n=48** |
| **1^st^ visit** | |  |  |  |  |  |  |
| Pain in the thigh, % (n) | | 16.7 (8) | 22.2 (10) | 24.2 (8) | 20.7 (6) | 24.5 (12) | 29.2 (14) |
| Pain in the lower leg, % (n) | | 6.3 (3) | 0.0 (0) | 0.0 (0) | 0.0 (0) | 2.0 (1) | 6.3 (3) |
| No pain in the leg, % (n) | | 39.6 (19) | 62.2 (28) | 78.8 (26) | 58.6 (17) | 59.2 (29) | 62.5 (30) |
| Never visited chiropractor for this problem before, % (n) | | 60.4 (29) | 57.8 (26) | 42.4 (14) | 58.6 (17) | 42.9 (21) | 47.9 (23) |
| Patients believe that their pain will get better 0-10 (No chance - Very likely), mean (SD) | | 8.45 (1.8) | 8.7 (1.5) | 8.5 (1.7) | 8.8 (1.7) | 8.3 (1.6) | 7.9 (2.2) |
| Lives alone, % (n) | | 6.2 (3) | 6.7 (3) | 3.0 (1) | 3.4 (1) | 0.0 (0) | 0.0 (0) |
| Pain severity (MPI) 0-6, mean (SD) | | 2.5 (0.9) | 2.7 (1.0) | 3.9 (0.7) | 3.4 (1.2) | 3.7 (1.0) | 4.0 (0.6) |
| Interference (MPI) 0-6, mean (SD) | | 1.9 (0.9) | 1.7 (1.1) | 3.3 (1.3 | 3.0 (1.4) | 3.7 (1.0) | 3.6 (0.8) |
| Life Control (MPI) 0-6, mean (SD) | | 4.2 (0.8) | 4.6 (0.9) | 2.4 (0.8) | 2.5 (1.1) | 3.2 (0.9) | 3.2 (0.7) |
| Affective distress (MPI) 0-6, mean (SD) | | 1.7 (1.0) | 1.4 (1.0) | 3.4 (1.3) | 3.4 (1.0) | 3.4 (0.9) | 3.3 (1.0) |
| Support (MPI) 0-6, mean (SD) | | 4.6 (1.3) | 4.3 (1.5) | 2.4 (1.5) | 2.3 (1.5) | 5.0 (0.7) | 4.8 (1.1) |
| Punishing responses (MPI) 0-6, mean (SD) | | 0.3 (0.5) | 0.3 (0.4) | 2.5 (1.8) | 1.8 (1.4) | 1.0 (0.8) | 1.1 (1.3) |
| Solicitous responses (MPI) 0-6, mean (SD) | | 2.4 (1.4) | 2.4 (1.2) | 1.5 (1.2) | 1.4 (1.0) | 3.7 (1.2) | 3.6 (1.1) |
| Distracting responses (MPI) 0-6, mean (SD) | | 2.4 (1.6) | 2.8 (1.6) | 1.9 (1.3) | 2.0 (1.2) | 3.7 (1.1) | 3.4 (1.1) |
| **4^th^ visit** | |  |  |  |  |  |  |
| Chiropractor believes that MC is appropriate for patient, % (n) | | 95.1 (39) | 97.6 (41) | 96.8 (30) | 100.0 (27) | 100 (45) | 100 (43) |
| Has taken analgesic medication for the pain, % (n) | | 15.6 (7) | 4.4 (2) | 21.2 (7) | 24.1 (7) | 20.4 (10) | 17.0 (8) |
| **Study start** | |  |  |  |  |  |  |
| Treatment given during the inclusion period % (n) | SMT/MOB/ACT/  DROP | 91.1 (41) | 97.0 (32) | 93.1 (27) | 93.9 (46) | 93.8 (45) | 93.9 (46) |
|  | STT | 68.9 (31) | 78.8 (26) | 65.5 (19) | 61.2 (30) | 66.7 (32) | 61.2 (30) |
|  | Information/  advice | 75.6 (34) | 93.9(31) | 79.3 (23) | 77.6 (38) | 68.8 (33) | 77.6 (38) |
|  | Other | 33.3 (15) | 33.3 (11) | 41.4 (12) | 22.4 (11) | 29.2 (14) | 22.4 (11) |
| Sick leave during the past year (at study start), % (n) | No sick leave | 92.5 (37) | 83.9 (26) | 88.9 (24) | 88.6 (39) | 74.4 (32) | 88.6 (39) |
|  | 1-7 days | 5.0 (2) | 16.1 (5) | 7.4 (2) | 9.1 (4) | 4.7 (2) | 9.1 (4) |
|  | 8-14 days | 2.5 (1) | 0.0 (0) | 3.7 (1) | 0.0. (0) | 9.3. (4) | 0.0. (0) |
|  | >15 days | 0.0 (0) | 0.0 (0) | 0.0 (0) | 2.3 (1) | 11.6 (5) | 2.3 (1) |
| Week 1, number of days with pain, n = 309, mean (SD) | | 2.3 (2.1) | 1.9 (1.8) | 3.3* (1.9) | 2.1* (2.0) | 2.3 (1.9) | 2.9 (2.2) |
