# Supplementary material for: The Nordic Maintenance Care Program: Does psychological profile modify the treatment effect of a preventive manual therapy intervention? A secondary analysis of a pragmatic randomized controlled trial
Source: PLoS One. 2019 Oct 10;14(10):e0223349. doi: 10.1371/journal.pone.0223349 (PMC6786625; doi:10.1371/journal.pone.0223349)
Supplement: S3 Table — A, no imputation has been made for missing data, mean values and percentages are based on true responses for each variable; MPI, West Haven–Yale Multidimensional Pain Inventory; MC, maintenance care; MOB, mobilization; ACT, mechanically assisted spinal manipulative therapy using the activator instrument or similar; DROP, mechanically assisted spinal manipulative therapy using a drop mechanism in table; STT, soft tissue treatment; ATM, use of active therapeutic movement treatment table; SD, standard deviation; n, number of subjects; RMDQ, Roland Morris disability questionnaire. (DOCX) [file pone.0223349.s003.docx]

| **Variable^A^** |  | **MPI sub-groups** | | | | | |
| --- | --- | --- | --- | --- | --- | --- | --- |
|  |  | **Adaptive Coper** | | **Interpersonally distressed** | | **Dysfunctional** | |
|  |  | **MC**  **n=48** | **Control**  **n=45** | **MC**  **n=33** | **Control**  **n=29** | **MC**  **n=49** | **Control**  **n=48** |
| Treatment given during the study period, % (n) | SMT | 93.8 (45) | 68.9 (31) | 81.8 (27) | 93.1 (27) | 91.8 (45) | 79.2 (38) |
|  | MOB/ACT/DROP | 29.2 (14) | 13.3 (6) | 36.4 (12) | 27.6 (8) | 32.7 (16) | 50.0 (24) |
|  | STT | 70.8 (34) | 51.1 (23) | 51.5 (17) | 65.5 (19) | 55.1 (27) | 56.3 (27) |
|  | ATM | 10.4 (5) | 4.4 (2) | 9.1 (3) | 17.2 (5) | 20.4 (10) | 8.3 (4) |
|  | Information/advice | 72.9 (35) | 51.1 (23) | 69.7 (23) | 72.4 (21) | 77.6 (38) | 54.2 (26) |
| Treatment by other health professionals, % (n) | Massage therapist | 10.4 (5) | 11.1 (5) | 12.1 (4) | 10.3 (3) | 4.1 (2) | 6.3 (3) |
|  | Chiropractor | 2.1 (1) | 0.0 (0) | 3.0 (1) | 3.4 (1) | 4.1 (2) | 0.0 (0) |
|  | Physiotherapist | 6.3 (3) | 8.9 (4) | 9.1 (3) | 27.6 (8) | 4.1 (2) | 14.6 (7) |
|  | Naprapath | 0.0 (0) | 4.4 (2) | 0.0 (0) | 3.4 (1) | 0.0 (0) | 2.1 (1) |
|  | Osteopath | 2.1 (1) | 0.0 (0) | 0.0 (0) | 0.0 (0) | 0.0 (0) | 2.1 (1) |
|  | Analgesic medication | 8.3 (4) | 4.4 (2) | 18.2 (6) | 13.8 (4) | 8.2 (4) | 12.5 (6) |
|  | Medical physician | 2.1 (1) | 2.2 (1) | 0.0 (0) | 10.3 (3) | 6.1 (3) | 6.3 (3) |
|  | Yoga | 2.1 (1) | 0.0 (0) | 0.0 (0) | 0.0 (0) | 0.0 (0) | 0.0 (0) |
|  | Acupuncture | 0.0 (0) | 2.2 (1) | 0.0 (0) | 0.0 (0) | 0.0 (0) | 0.0 (0) |
|  | Laser | 0.0 (0) | 2.2 (1) | 0.0 (0) | 0.0 (0) | 0.0 (0) | 0.0 (0) |
| Sick leave during the past year, % (n) | No sick leave | 91.7 (44) | 82.2 (37) | 75.8 (25) | 65.5 (19) | 71.4 (35) | 68.8 (33) |
|  | 1-7 days | 0.0 (0) | 4.4 (2) | 18.2 (6) | 10.3 (3) | 14.3 (7) | 12.5 (6) |
|  | 8-14 days | 2.1 (1) | 2.2 (1) | 0.0 (0) | 3.4 (1) | 2.0 (1) | 4.2 (2) |
|  | >15 days | 0.0 (0) | 0.0 (0) | 0.0 (0) | 0.0 (0) | 6.1 (3) | 4.2 (2) |
| Patient feels care plan is worth continuing with, % (n) | Definitely worth it | 54.2 (26) | 46.7 (21) | 57.6 (19) | 44.8 (13) | 57.1 (28) | 41.7 (20) |
|  | Possibly worth it | 29.2 (14) | 26.7 (12) | 24.2 (8) | 10.3 (3) | 24.5 (12) | 22.9 (11) |
|  | Undecided | 2.1 (1) | 6.7 (3) | 9.1 (3) | 13.8 (4) | 8.2 (4) | 12.5 (6) |
|  | Hardly worth it | 8.3 (4) | 8.9 (4) | 3.0 (1) | 6.9 (2) | 4.1 (2) | 2.1 (1) |
|  | Definitely not worth it | 0.0 (0) | 0.0 (0) | 0.0 (0) | 0.0 (0) | 2.0 (1) | 8.3 (4) |
| Pain intensity 0-10, mean (SD) |  | 1.93 (1.97) | 1.32 (1.70) | 2.39 (2.17) | 2.26 (2.32) | 1.96 (2.11) | 2.35 (2.35) |
| EQ5D score 0-1, mean (SD) |  | 0.87 (0.08) | 0.89 (0.09) | 0.85 (0.09) | 0.85 (0.08) | 0.85 (0.12) | 0.79 (0.20) |
| EQ5D difference (follow up - baseline), mean (SD) |  | 0.05 (0.09) | 0.10 (0.13) | 0.23 (0.23) | 0.19 (0.22) | 0.26 (0.27) | 0.15 (0.24) |
| Health in general at (follow up, mean (SD) | Excellent | 14.6 (7) | 13.3 (6) | 15.2 (5) | 3.4 (1) | 6.1 (3) | 12.5 (6) |
|  | Very good | 47.9 (23) | 51.1 (23) | 24.2 (8) | 37.9 (11) | 46.9 (23) | 31.3 (15) |
|  | Good | 25.0 (12) | 22.2 (10) | 45.5 (15) | 34.5 (10) | 34.7 (17) | 27.1 (13) |
|  | Quite poor | 6.3 (3) | 2.2 (1) | 9.1 (3) | 3.4 (1) | 8.2 (4) | 16.7 (8) |
|  | Poor | 0.0 (0) | 0.0 (0) | 0.0 (0) | 0.0 (0) | 0.0 (0) | 2.1 (1) |
| RMDQ Score 0-24, mean (SD) |  | 2.95 (2.91) | 2.91 (4.14) | 4.15 (3.34) | 3.95 (4.16) | 3.58 (4.41) | 4.23 (4.90) |
| RMDQ difference (follow up - baseline), mean (SD) |  | -0.72 (4.01) | -0.35 (3.98) | -2.26 (3.62) | -0.64 (2.48) | -2.59 (3.68) | -1.58 5.34) |
| Pains effect on productivity during past month 0-10 (Did not affect work - Prevented work completely), mean (SD) |  | 1.11 (1.37) | 1.18 (1.60) | 1.81 (1.76) | 1.64 (1.97) | 2.02 (2.03) | 2.95 (2.62) |
